# Video Recording and Analysis of Avian Movements and Behavior: Insights from Courtship Case Studies

**DOI:** 10.1093/icb/icab095

**Published:** 2021-05-25

**Authors:** Judith Janisch, Clementine Mitoyen, Elisa Perinot, Giovanni Spezie, Leonida Fusani, Cliodhna Quigley

**Affiliations:** 1 Konrad Lorenz Institute of Ethology, University of Veterinary Medicine, 1160 Vienna, Austria; 2 Department of Cognitive Biology, University of Vienna, 1090 Vienna, Austria; 3 Vienna Cognitive Science Hub, University of Vienna, 1090 Vienna, Austria

## Abstract

Video recordings are useful tools for advancing our understanding of animal movements and behavior. Over the past decades, a burgeoning area of behavioral research has put forward innovative methods to investigate animal movement using video analysis, which includes motion capture and machine learning algorithms. These tools are particularly valuable for the study of elaborate and complex motor behaviors, but can be challenging to use. We focus in particular on elaborate courtship displays, which commonly involve rapid and/or subtle motor patterns. Here, we review currently available tools and provide hands-on guidelines for implementing these techniques in the study of avian model species. First, we suggest a set of possible strategies and solutions for video acquisition based on different model systems, environmental conditions, and time or financial budget. We then outline the available options for video analysis and illustrate how different analytical tools can be chosen to draw inference about animal motor performance. Finally, a detailed case study describes how these guidelines have been implemented to study courtship behavior in golden-collared manakins (*Manacus vitellinus*).

## Introduction

Our understanding of animal behavior often depends on our ability to precisely quantify movement. The way animals move in space is critical for survival (locomotion for foraging, feeding, or fleeing) and reproduction (courtship to obtain copulations), and specific changes in body position, configuration, speed, or orientation can be linked to adaptive values in different model systems. Measuring movement comes with many challenges, including environmental factors, the size and speed of the animal or body parts of interest, and the spatial scope of the target behavior. To advance our understanding of the functional significance of behavior, we need to choose the right tools.

Much effort has been devoted to the development of tools to quantify motor behavior. Apart from bio-loggers that measure spatial location or orientation (Fehlmann and King 2016), one of the most frequently used tools is video recording. Compared with direct observational coding, video allows for repeated analysis of the same behavior by different researchers, as well as inspection of recorded videos at different speeds and spatial resolutions to reveal behaviors that are too fast or small for human eyes (Noldus et al. 2002; Egnor and Branson 2016). The introduction of high-speed video recordings with a temporal resolution of hundreds or even thousands of frames per second has enabled the study of very rapid behaviors, including wing sonation mechanisms in manakins (Bostwick and Prum 2005; Bodony et al. 2016) and foot-tapping in blue-capped cordon-bleu (*Uraeginthus cyanocephalus*) (Ota et al. 2015).

Another important advance for describing animal movements was the introduction of motion capture systems. These systems enable precise tracking of pre-defined points in 2D or 3D to study locomotion and biomechanics. They often use physical markers, typically placed on the limb joints of the moving animal. For example, markers have been used in pigeons (*Columba livia*) to study wing and body kinematics during take-off and landing (Berg and Biewener 2010) and body orientation during flight (Ros et al. 2011). Recent advances in markerless motion capture and tracking are of particular interest for the study of animal behavior, as markers may alter an animal’s movements (e.g., impaired aerodynamics) or behavior (e.g., changed appearance affecting mating success).

Courtship displays represent some of the most fascinating and elaborate behaviors and often include incredibly fast or subtle movements. Birds in particular have evolved a huge variety of courtship displays. For example, during the breeding season, duetting couples of grebes coordinate their postures and gestures in an elaborate courtship dance (Nuechterlein and Storer 1982). Male manakins exhibit extremely demanding acrobatic displays individually or cooperatively (e.g., DuVal 2007; Fusani et al. 2007), including red-capped manakins (*Ceratopipra mentalis*) that perform a “moonwalk” to impress females, and the blue manakin (*Chiroxiphia caudata*) where several males dance together (Kirwan and Green 2011; Brodt et al. 2014; Fuxjager et al. 2016). The ability to execute vigorous or highly coordinated body movements during courtship is selected for by females in a variety of species (Borgia and Presgraves 1998; Patricelli et al. 2002; Byers et al. 2010; Fusani et al. 2014). To fully understand the link between motor performance and reproductive success, or to gain insights into the proximate mechanisms underlying complex motor patterns, it is important to identify features of motor performance that undergo sexual selection and to measure them.

**Fig. 1.**
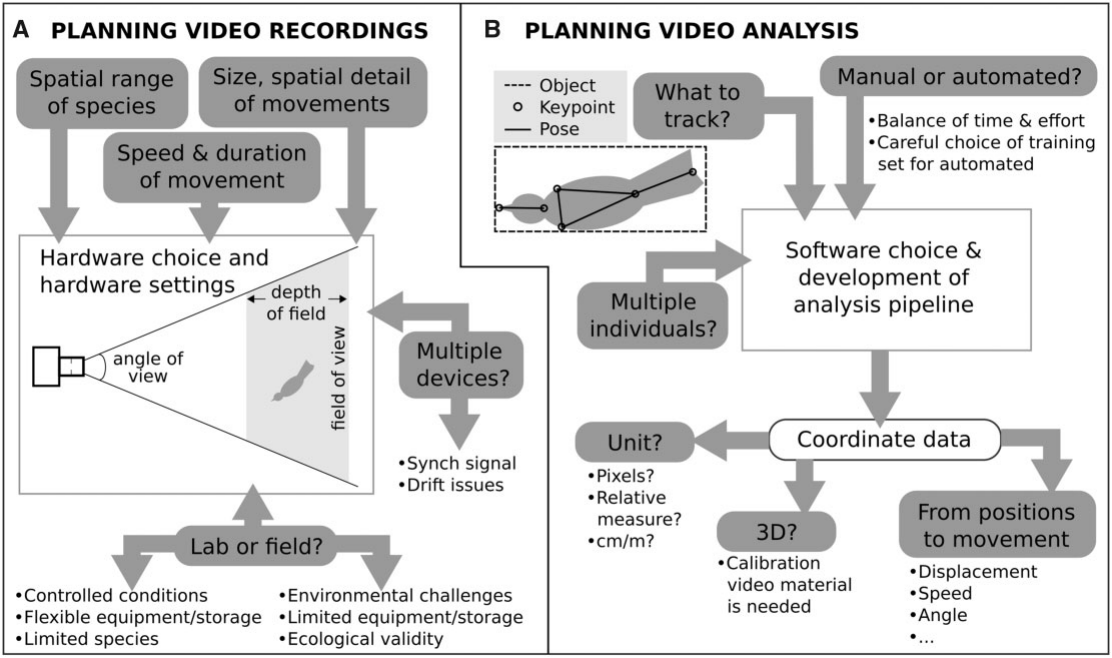
Visualization of the decisions involved in planning video recording and analysis to quantify animal movements. (**A**) Multiple factors impact the choice of hardware and hardware settings that should be used to optimally record the chosen study species in the context of the current research question. (**B**) The choice of video analysis software is also impacted by several factors. The end-point of the analysis is typically spatial coordinate data which can be further analyzed in different ways.

In this article, we discuss key issues in the use of video recordings to study elaborate and complex motor behavior in laboratory and field conditions and provide some general guidelines (summarized in Figure 1). We focus on courtship displays of birds because our group has been studying these behaviors for the last three decades. Generally, avian courtship displays are very diverse and often involve very elaborate movement patterns, and can be representative of the types of challenges encountered when studying behavior of many taxa. The use of videos for recording and analyzing movements of many individuals (collective behavior) has been covered by previous reviews (e.g., Hughey et al. 2018) and is not considered here. The section “Video recordings: planning data collection” discusses various factors that should be considered before deciding on a recording system for 2D or 3D video, keeping in mind the limitations and problems in field and laboratory conditions. The section “Post-processing video material you have collected” addresses various options that are available for video analysis, including automated techniques. Case studies investigating courtship displays in a few bird species are used as examples throughout, and the section “Case study: 3D motion capture in golden-collared manakins” contains a detailed case study of 3D field recordings in golden-collared manakins (*Manacus vitellinus*). We conclude with general remarks on the future possibility of fully automated analysis of animal behavior using video recording and analysis of animal movements.

## Video recordings: planning data collection

A video recording system typically includes camera(s), lens(es), lighting, and a data storage medium. Video recording equipment that is suitable for capturing animal movements ranges from stand-alone consumer-grade cameras to completely custom-made set-ups including dedicated lighting and multiple computers (see Table 1 for examples). We list the main components in Text Box 1, but a detailed description of the parameters involved in camera sensors and optics is beyond the scope of this article (for a general introduction, see Loopbio 2021; Nikon 2021). In this section, we examine requirements in light of the physical and behavioral features of the target species, namely, spatial extent, size, speed, number of focal individuals, and whether multiple recording modalities are required.

**Table 1.** Summary of different case studies investigating movements of birds during courtship

Study species	Ring dove	King penguin	Spotted bowerbird	Golden-collared Manakin
Study cited	Mitoyen et al. (2021)	Mitoyen et al. (manuscript in preparation)	Spezie et al. (manuscript in preparation)	Janisch et al. (2021)
Study question	How do females respond to male courtship parameters?	How do courtship parameters influence pair formation and reproductive success?	How does courtship intensity relate to audience behavior?	How do courtship movements differ between individuals?
Variables measured	Timing of male bowing display, bowing amplitude	Timing of male and female movements, amplitude of head raises	Crouching behavior, amplitude of receivers’ movements	Trajectories of jumps used to derive initial velocity, acceleration, and force
Recording environment	Laboratory	Field	Field	Field
Spatial range of behavior	Restricted to experimental set-up	Gather in colony of thousands of individuals	Males defend an arena, on which they build and decorate a bower; receivers are inside the bower	Males maintain an arena, where they jump between saplings
**Video recordings**				
Recording type	2D	2D	2D	3D
Cameral model	Basler acA1920-155uc	Black Magic Pocket camera	Browning Record Force Advantage 2018 HD	Basler acA1920-155uc
Number of cameras per individual	2	1	1	3
Camera mounting	Fixed on aluminium frame	Mobile and portable, tripod mounting	Fixed on tree or tripod	Semi-mobile, fixed on tripods during recording sessions
Camera settings	Fixed manual exposure time, fixed aperture and focus	Autofocus and autoexposure	Autofocus and autoexposure	Fixed aperture and focus, manual change of exposure time
Control of recordings	Web interface from desktop computer in lab	On the camera directly	Camera trap triggered by movement detection	Web interface from mobile phone or tablet
Recording frame rate	60	30	30	60
Audio recordings	Yes, camera hardware synchronizer recorded as audio input to allow audiovisual synchronization	Yes, camera has external microphone input	Yes, inbuilt microphone	Possible, but not used
Synchronization of multiple recordings	Hardware synchronizer	Audiovisual synchronization dealt with by camera	Audiovisual synchronization dealt with by camera	Hardware synchronizer
**Video processing**				
Annotation type	Automated keypoint tracking (head, beak, feet, and tail)	Hybrid manual/automated depending on presence of other individuals, keypoints (head, beak, and flippers)	Automated keypoint tracking (beak, eyes, tail, feet, and wing crease)	Automated object detection with bounding box
Number of training frames for automated tracking	1,000	<500	<1,500	150,000
Choice of training frames	Training frames encompassed the full range of plumage color and different behavioral states of the experimental birds	Balanced number of frames based on distance from the camera and frame background (empty environment vs. colony)	Balanced number of frames based on lighting conditions and contrast, distance of bird(s) from camera, bird orientation in relation to camera	Single field season, all videos manually annotated

Video examples are contained in the Supplementary Material.

**Text Box 1** Glossary of terms related to video recording systems

**Aperture**: Size of the opening in the camera lens; determines the amount of light falling on the image sensor; influences the depth of field (larger opening results in shallower depth of field) and motion blur.

**Codec**: Scheme for encoding and decoding digital video. Most codecs involve “lossy” data compression, typically tailored to human perception and discarding any information that is imperceptible to humans at normal viewing speed. Compression may influence the outcome of later video analysis.

**Depth of field**: The portion of space that will be rendered in sharp detail in the recorded image; determined by the aperture and other lens properties.

**Exposure**: The total amount of light falling on the image sensor during the acquisition of each frame; determined by aperture and shutter speed.

**Focus**: In zoom lenses, the focal length can be changed, which affects the distance from the camera which will be rendered sharpest in detail.

**Frame rate**: the number of images acquired by the camera per unit time; usually defined as frames per second (fps). The standard rate used in cinema films is 24 fps.

**Gain**: Available in some recording systems, gain allows for an increase in the amplitude or brightness of the acquired image; also increases any noise present in the image.

**Lens**: Focuses light onto the camera’s image sensor; main property that can be altered is aperture, and focus for zoom lenses. Depending on the camera, interchangeable lenses may be used.

**Shutter speed**: determines the duration used to acquire a single image; also known as exposure time. Longer durations require less ambient illumination, while short durations are best for capturing fast-moving objects.

**Spatial resolution**: the physical size of the acquired image in pixels; higher resolution images require more memory for file storage.

### Spatial range of movements of interest

Lens properties including focal length and aperture determine the angle of view and depth of field (see Text Box 1), and objects that are located outside the depth of field will not be in focus in the recorded image. Video recording systems are therefore particularly useful when the movement of interest is spatially limited and repeatedly performed within the same space, as in animals that possess territories or perform courtship displays in leks—display grounds where males gather to compete for females (Höglund and Alatalo 1995). The dimensions of the space where the movements of interest occur, together with the distance the camera can be placed from the focal animal, are important factors when choosing hardware (e.g., camera spatial resolution and lens).

For example, birds of paradise like the Carola’s Parotia (*Parotia carolae*) who construct and maintain display courts have been filmed from behind blinds located close to their courts (Scholes and Sodhi 2006). In some cases, it is advantageous to set up a camera trap, which is a fixed system that can be automatically triggered by movements (see Supplementary Table S1) and can be mounted on a tripod or fixed to a tree. Camera traps have been used to target bowerbirds (Fam. Ptilonorhynchidae) for entire breeding seasons, with recordings controlled by a movement-triggered sensor (Borgia 1995a). These birds build complex structures—known as bowers—which are visited by females and are used as part of an elaborate courtship display (Borgia 1995b; Frith and Frith 2004). When using motion-activated cameras, it is crucial to understand the consequences of the parameters specific to camera traps, namely video trigger and recovery speed. These parameters can potentially affect data collection, especially when the behaviors of interest are rapid, such as copulations. We recommend validating the camera settings by carrying out direct observations (e.g., Madden 2003) or using a second camera which records continuously. Some camera trap models continue to record during motion detection, overriding video length settings (spotted bowerbird case study in Table 1).

In contrast, some species do not restrict their behavior to a clearly delimited area or their courtship ground is not predictable. An example is King penguins (*Aptenodytes patagonicus*), where courting couples are highly mobile, sometimes travelling dozens of meters between episodes of a full courtship interaction (Jouventin and Dobson 2017). In this case, the experimenter can follow the displaying individuals while carrying a mobile recording set-up. For example, a lightweight camera with variable focal length can be transported in a backpack and quickly mounted on an adjustable tripod when needed (penguin case study in Table 1).

At the other extreme, laboratory-based video recordings can allow complete experimental control of the volume of space in which the animal moves and therefore equipment is typically fixed in position (dove case study in Table 1). In a study by Fischer et al. (2020), for example, the authors recorded the courtship of the false black widow spider (*Steatoda grossa*), while the animals were housed in plexiglass boxes with lighting conditions optimized to enhance image quality. Laboratory conditions also allow more control over which individuals appear in recordings, making post-processing of videos easier.

### Size of species and level of spatial detail needed

The size and morphology of the model organism will also influence hardware choices. In particular, the choice of the lens and its focal length, and the spatial resolution of the recordings will determine whether the body parts of interest are captured in sufficient detail to quantify subtle movements. For example, Dankert et al. (2009) measured the subtle and fast movements of courtship behavior in *Drosophila melanogaster*, and the spatial resolution of the cameras was crucial to allow visualization of the movements of single body parts including wing position.

However, if the study hypotheses focus on the movement trajectory of the entire animal, it is not necessary to track specific body parts. For example, in the case study on manakins below (see “Case study: 3D motion capture in golden-collared manakins” section), we chose to track the whole individual as a single object. This allowed us to use a lower spatial resolution and frame rate, which kept file sizes smaller. This aspect is particularly important in field settings.

### Speed of movements

An additional factor to be considered is the speed of the movement of interest. For motion of any given speed and duration, the frame rate of the video must be high enough to capture sufficient details for later analysis. For very brief events, the duration of an individual frame must be less than the full duration of the movement of interest. For example, the roll snaps (clapping wings together behind their back) of golden-collared manakins happen at a rate of 60 Hz, corresponding to a duration of <16 ms, and a frame rate of 2,000 fps was required to study this movement in detail (Bodony et al. 2016). The male of *C. capitator* performs a behavior called wing-fanning (fluttering his wings facing a female) at >200 Hz and the authors used 1,000 fps to record the behavior (Benelli et al. 2020).

Although frame rates available in standard action cameras are relatively fast (up to 240 frames in entry-level models, see Supplementary Table S1), recording at high frame rates comes with several associated costs. First, higher frame rates typically involve a trade-off in spatial resolution of the recordings, as computational limits are quickly reached regarding the amount of data that can be acquired, processed, and saved in the short amount of time available between frames. Second, an increase in frame rate reduces the time available for exposure of each composing frame, which can be a limiting factor when recording in low environmental light. Finally, the file size of a video is proportional to the frame rate and can lead to issues with data storage, transfer, and backup.

As well as frame rate, shutter speed and aperture are crucial factors to consider whenever later analysis will require crisp images of fast-moving objects. Fast shutter speeds are necessary to avoid motion blur, as each image will contain as little movement as possible, providing the best estimate of the current location of the animal or configuration of the body part of interest (see Pueo 2016 for an in-depth discussion of this topic). The trade-off between increasing exposure time to improve color resolution and reducing it to limit motion blur is an important constraint for field recordings, especially where lighting conditions are changeable. Even in laboratory conditions, artificial lighting needs to be carefully evaluated to avoid impacting the animals’ natural behavior. For example, Fischer et al. (2020) used a white-fluorescent light to improve image quality while recording false black widow spider courtship, as previous studies showed the spiders’ behavior to be unaffected by it. Fast moving animals that move over large distances relative to their body size present an extreme challenge. A compromise can be made by using a grayscale camera sensor that acquires luminance only and not color information, thereby boosting the available spatial resolution (e.g., Bodony et al. 2016).

### Laboratory studies

Some bird species can be kept in captivity and used in laboratory studies of courtship. Although behavior in the laboratory sometimes differs to natural conditions (Egnor and Branson 2016), more controlled experimental designs can be used, and video-based data can be constrained in more respects than field recordings, making later processing and analysis easier. For example, the use of controlled lighting and a uniform colored background allow high-contrast recordings of focal individual(s) (Branson et al. 2009; Mitoyen et al. 2021) which can greatly facilitate tracking of individuals or body parts. It is important to note that regular room lighting should not be used when working with birds for welfare reasons (Joint Working Group on Refinement 2001), as they perceive flickering of lights that is imperceptible to humans. In addition, this flickering is often an issue in video recordings, depending on the combination of video frame rate and domestic alternating current rate (50 Hz in Europe and 60 Hz in the USA). For both reasons, it is best to use lighting powered by a direct current source for laboratory video recordings when working with birds. Studio lighting such as LED softboxes can be used for diffuse lighting, while for targeted lighting, LED spotlights can be fixed on multi-directional mounts (Mitoyen et al. 2021).

Additionally, in the laboratory, there is often no limitation regarding available electrical power, disk space, and computing power. For example, the EthoLoop framework (Nourizonoz et al. 2020) uses up to six cameras and graphical processing units (GPUs) to perform real-time 3D marker-based tracking of up to three individuals moving in a 3D behavioral arena and additionally includes a mobile high-resolution camera that follows a focal individual to detect target behaviors and control a behavior-contingent reward system.

### Environmental challenges in field recordings

When recording video in the field, or under semi-natural conditions such as large aviaries in the forest, several additional aspects should be taken into account during planning. First, access to electricity might be limited, meaning it is necessary to work with batteries. Some stand-alone camera systems (e.g., action cameras or camera traps, Supplementary Table S1) have internal rechargeable batteries or connections for solar panels and can be used without direct electrical power during recordings. In general, the energy demands of video recordings depend on the duration, spatial resolution, and frame rate, hence battery life is inversely proportional to recording quality. These are important parameters to consider and, if possible, to test before beginning recordings. Camera trap batteries, which can last for months when used in photo mode, only last a few days when recording videos, which are essentially stored as a series of pictures with time stamps. As a rough guideline, videos use up as much memory storage and battery power as would the number of video frames (duration times frame rate) if considered as photos. For example, a battery capable of powering a Browning Recon Force Advantage 2018 HD to take 60–80 photos per day over many months will last only 2–3 days when set to take videos of 30–120 s at 60 fps. For more sophisticated recording systems that include a computer component, motorbike or car batteries are a good and cheap alternative that can be recharged between recording sessions.

The weight of the equipment is important, particularly if it has to be regularly moved between recording sites or carried for long distances. Environmental and weather conditions also play a vital role in choosing a recording system. Most devices will specify a range of operating temperatures, but there can be temperature-dependent variations in performance even within the operating range. Heat or cold, rain, sand- or snowstorms, and other adverse conditions can easily damage or destroy equipment. When working in the tropics, for example, it is a good idea to use waterproof cameras or at least have a quick and easy means of covering them adequately during rain. In hot and humid climates, problems with overheating and internal humidity can occur and in the worst case can halt operation during data acquisition. Internal fan ventilation in computer systems is the best way of avoiding temperature problems, but this has the drawback of being noisy, possibly disturbing animals during recordings. In general, it is worth placing any electrical equipment so that it has optimal airflow around it, for example, raising a recording computer off the ground.

While recording, short-term environmental changes such as moving background elements and general ambient lighting conditions can be highly variable and often unpredictable. The use of as large a depth of field as possible, and automatic focus setting is almost mandatory in field conditions, as the distance of the focal individual(s) from the camera can change rapidly, making changes in a manual focus setting impossible to perform. The same is true of exposure, as the amount of available light may change over the course of a recording, either gradually or suddenly. If possible, automatic exposure should be enabled, particularly if the movements and not the visual appearance of the focal bird are important. In recording systems where automated settings for focus and exposure are not available, and particularly in recording systems where the cameras cannot be physically accessed by the experimenter, the ability to remotely change related parameters is highly advantageous (see “Case study: 3D motion capture in golden-collared manakins” section). Finally, wild animals can roam around freely and can move outside the field of view of the camera at any time, or become occluded by other elements in the environment (Egnor and Branson 2016). Direct focal observations or additional wide-angle cameras can be extremely helpful to keep track of events happening outside the recorded area.

### Simultaneous multi-device recordings

**Fig. 2.**
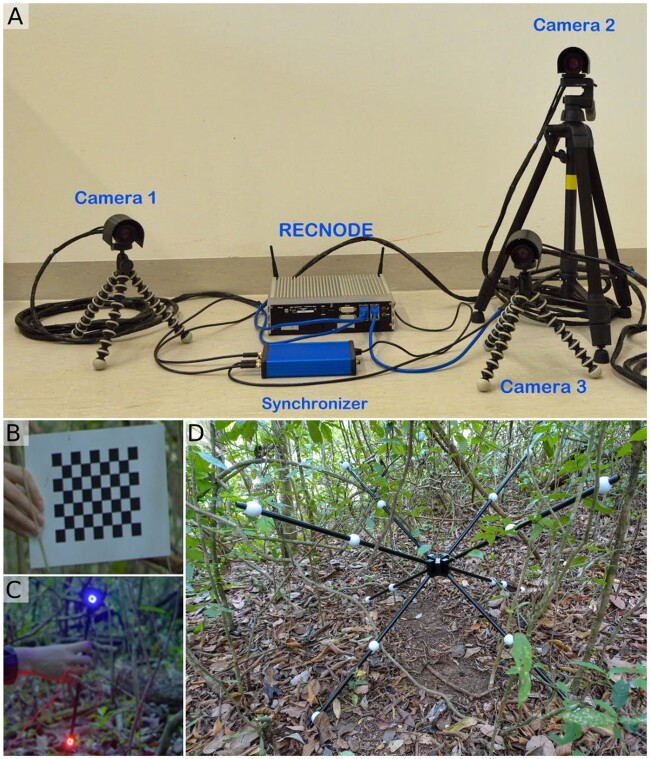
Example multi-camera recording system. (**A**) The three cameras on tripods are connected to a central computer (“Recnode”) and a hardware synchronizer. (**B**) Checkerboard used for intrinsic calibrations and (**C**) Wands with two different light sources used for extrinsic calibration of cameras. (**D**) Multi-armed structure for ground-level calibration, with known distances between white balls and a spirit level in the center (see section “Camera calibration” for more detail).

Depending on the research question, it may be necessary to record from more than one camera or additional devices like microphones. Whenever multiple recordings are made simultaneously, it is important to either synchronize them or to measure the relative timing of the different recording devices. A hardware synchronizer directly triggers the acquisition of each frame by each camera (Figure 2). In our dove recordings, for example, we route the camera synchronization trigger signal into an audio mixer, where it is recorded as an extra audio track, allowing video and audio recordings to be synchronized in post-processing (Mitoyen et al. 2021). Some cameras also include an output stream of frame-triggered pulses which can be recorded and used for later synchronization (e.g., Bodony et al. 2016 used the output of a high-speed camera to synchronize audio recordings). Open-source alternatives to hardware triggers are possible, for example, Jackson et al. (2016) used walkie–talkies to provide simultaneous audio input to commercial action cameras, and Laurijssen et al. (2018) used a combination of a blinking LED and a bit-stream recorded as an additional audio channel. Alternatively, a simple (audio) visual alignment signal can be created using a clapperboard or by clapping hands in front of the camera.

It is important to keep in mind that different hardware devices may differ in their internal clocks. Even if there is only a small, sub-millisecond difference in their timing, this will accumulate over time and the recordings will drift apart. The use of a hardware trigger signal is extremely advantageous in such cases, as the onset of each video frame is temporally marked, allowing recovery of synchronization for later time-points of interest in a long recording. Alternatively, a clapperboard can be used to mark at least two time-points (e.g., at the start and end of each recording) to provide an estimate of drift to compensate for it in post-processing.

Sometimes the use of multiple cameras to simultaneously record video from different viewpoints may solve otherwise very difficult challenges. For example, in our laboratory recordings of ring doves (dove case study in Table 1), male bowing movements during courtship are well characterized in single videos, because the male typically bows along an axis parallel to the image plane captured by one camera. However, we cannot easily estimate which direction the female is looking using a single camera. For this reason, we simultaneously recorded from a camera above the testing compartment, which we can use to determine head direction.

Recording more than one individual adds a level of complexity to later video analysis, as they might cross paths or visually occlude each other. For some behaviors, it might even be advantageous to record with multiple cameras to recreate the birds’ movements in 3D, which will be discussed in detail in the last section “Case study: 3D motion capture in golden-collared manakins” If the movement can be adequately described in 2D (or using multiple 2D views), then 3D recordings are probably not necessary.

### Budget and time planning

The planning of any project is subject to budget constraints. For video recording equipment, options range from bespoke systems available from companies specialized in recordings of animals, to the commercial equipment preferred by amateur sports video producers, through to lower budget, open-source solutions (see Supplementary Table S1). In general, more expensive options will require less time and technical know–how on the part of the researcher. Planning of data storage and backup is also extremely important, as large file sizes mean that many hard drives may be needed to store field data.

The equipment used can have an impact on the time plan of any project. Therefore, planning should consider the training needed for researchers to be able to acquire videos of high enough quality for later analysis. Time-intensive activities during recording include the set-up of mobile recording systems (e.g., consider camera traps to save time) or downloading and copying video material at the end of the day (e.g., consider internet access at the field station). In general, it is always good practice to look through recordings as often as possible during the entire recording phase of the project to check their quality and content.

## Post-processing video material you have collected

The increasing temporal and spatial resolution of recording systems has the consequence that extremely large amounts of data are generated when recording animal movements. We do not cover issues related to video file types and codecs, or sensible file-naming conventions here, but note that these are important. In this section, we cover some of the options available for processing and analyzing video data.

### Pre-screening of video recordings

Before starting data analysis, recordings should be checked to confirm the intended content and quality. Some systems, such as automatically triggered camera traps, produce substantial amounts of unwanted video material due to false triggers (e.g., strong wind causing moving vegetation, local wildlife, or behaviors that are not relevant to the specific research questions). In our example case of fixed camera traps positioned at bowers of spotted bowerbirds (bowerbird case study, Table 1), ∼80% of recorded video files did not contain relevant content (Spezie et al. manuscript in preparation). Thus, while motion-activated cameras provide the advantage of filming multiple individuals simultaneously (therefore, optimizing time budget during video acquisition), false triggers may translate into days of additional screening work. Software is available to reduce the manual labor associated with screening camera trap recordings (see Swinnen et al. 2014; Weinstein 2015). These algorithms analyze frame-to-frame variation in pixel values, identifying frames and recordings of interest using pre-specified thresholds for filtering motion events (see Supplementary Table S2 for further options). A further task might be to note which individuals are present in each video, which is particularly challenging when recording group or colony living birds. Marking animals beforehand can be very advantageous in these cases. In birds, this is typically done with colored rings, but larger marks are also sometimes needed. Automated methods have been proposed for identifying unmarked individual birds in recorded videos (Ferreira et al. 2020; see also Kühl and Burghardt 2013), which are based on the machine learning methods we describe below.

### Manual versus automated approaches to describe movements

When the final collection of relevant video recordings has been defined, the next step is to label the entire animal or the body part(s) of interest in the frames of interest. Manual annotations of large amounts of video data can be very time consuming, and automated annotation approaches are becoming more established (Valletta et al. 2017). Once appropriately validated (e.g., Janisch et al. 2021), automated approaches yield more rigorous and reproducible results, which are free of human errors and biases resulting from manual annotations. These methods (see Supplementary Table S2 for a list of available software) typically use machine learning algorithms that implement supervised learning techniques, meaning that a set of training data must be prepared by the experimenter using manual annotation. The algorithm will learn to detect whatever visual features the experimenter has annotated in the training data, so careful selection of representative video frames is needed (Valletta et al. 2017). In contrast, unsupervised learning does not require labeled training data and instead learns the statistical structure of the data set from the data itself. Although unsupervised methods may be useful in future, examples are rare even for simplified, low-dimensional video sets (e.g., Klibaite et al. 2017 applied such a technique to thresholded high-contrast recordings of courting flies) and it is not clear whether current approaches will work for high-dimensional data like that contained in color video recordings with complex backgrounds (Todd et al. 2017).

While automatic methods can increase efficiency and repeatability, manual annotations may be more efficient than automatic tracking under particular circumstances. For example, in colonial species gathering in their hundreds or thousands, it might simply not be possible to efficiently train a model to automatically track individual(s) of interest as the trained model may fail to consistently track the visual features of interest on target individuals filmed on a background of many similar individuals. Additionally, automatic annotation of a full sequence of movement (i.e., the annotation of every frame of a given video) might not be necessary depending on your research question. In both cases, manual annotation can be a more accurate and efficient solution. The time required to manually annotate training images, train and validate the algorithm, and then process the resulting coordinates (typically for all the frames of the video) might in some cases be longer than manually annotating only the points of interest at the times of interest. For example, when recording King penguin courtship in the field (penguin case study, Table 1), Mitoyen et al. (manuscript in preparation) were interested in the relative amplitude of head raises performed by females and males during courtship. To calculate the amplitude, solely the starting and the ending positions are necessary. Given that the rate of those raises is rather slow (a cycle takes at least 20 s), developing automatic annotation of the movement did not represent a significant gain of time. Thus, depending on the specific research question it is worth considering whether manual annotation is sufficient to derive the movements of interest.

### Different aspects of motion analysis: object detection, keypoint estimation, and pose detection

Based on different research questions, focus can be put on different aspects of an animal’s motor performance (see Figure 1). First, quantifying the movement of an animal as a whole to understand how one or more animals move in space (i.e., direction, trajectory, or speed) can be achieved using object detection. The manakin case study below provides a detailed example where automatic object detection was used to investigate the parabolic trajectory of jumps (Janisch et al. 2021). In this case, a bounding box marked the spatial limits of the entire bird, and its center of mass was used to estimate location in each video frame. Object detection is advantageous in this case as the fast-moving bird of interest is often slightly blurred on each video frame, making it hard to visually distinguish body parts.

Second, detecting and tracking movements of specific points on an animal’s body is referred to as keypoint estimation. When an animal is stationary, keypoint coordinates can be used to calculate the movement of single body parts relative to the whole body. This is useful in courtship displays in which coordinated movements of single body parts (known as gestures; Kendon 2004; Tobiansky et al. 2020) are produced to attract mates. For example, Fusani et al. (1997) described the bowing movements of courting male ring doves (*Streptopelia risoria*) using the change over time in *y* coordinates of the eye relative to the foot.

Third, it is possible to track the relative movements of different body parts, and the different configurations of body parts which result from their relative movement are referred to as “pose.” Pose estimation may help investigate actions that require particular coordination and precision, for example, foraging techniques (Voelkl and Huber 2007) and nut-cracking behavior in primates (Liu et al. 2009). In the context of courtship, simultaneous actions involving different body parts and the degree of precision in the control of those movements have a strong adaptive value, as females have been shown to pay attention to subtle differences in movement parameters, although in a narrow range of species (Backwell et al. 1998; Murai and Backwell 2006; Perez and Backwell 2020). Computational tools (see Supplementary Table S2) can be used to quantify fine-grained differences in pose expression and may shed light on the factors underlying mate choice. Avian species which exhibit particularly rich repertoires of courtship moves (e.g., bowerbirds, bird of paradise, and manakins) would profit from this approach, as the role that body configuration plays in defining attractiveness has so far been overlooked in sexual selection studies, which mostly focus on speed and other correlates of vigor. The analysis of pose, therefore, represents a fruitful avenue for future research.

### Simultaneous tracking of multiple individuals

It may be the case that the movement of interest concerns more than one individual. For example, in avian courtship, duet dances are widespread among monogamous species (Nuechterlein and Storer 1982; Malacarne et al. 1991; Ota et al. 2015; Soma and Iwama 2017); and in a variety of polygynous species, females actively participate in the courtship routine. These model systems provide potential for investigating the reciprocal influence of movement on courtship behavior, namely how two “objects” move with respect to one another, or whether specific visual cues from females may be followed by changes in male display structure.

Automated methods can be used for tracking multiple individuals simultaneously by assigning distinct identifiers for supervised learning (e.g., Pérez-Escudero et al. 2014; Walter and Couzin 2021, see Supplementary Table S2), although a few caveats need to be taken into account. First, when individuals are unmarked and visually similar, models may fail to assign the correct identifiers. Whenever the individuals are very close or cross paths in the recorded image, their identifiers may be swapped, as most current algorithms do not integrate tracked movement over time in the way a human observer does (for recent advances, see Supplementary Table S2). Conversely, automatic tracking of multiple individuals typically succeeds when one of the two individuals moves within specific boundaries of the field of view (e.g., within the bower walls in bowerbirds), when their relative position is fixed (two king penguins courting side by side, e.g., Jouventin and Dobson 2017, or two java sparrows courting on a fixed branch, e.g., Soma et al. 2019), or when dimorphism in color or body morphology allows a more obvious distinction of individual identities (Mulder 1997; Scholes 2008; Spezie et al. manuscript in preparation). One possible solution for sexually monomorphic individuals are markers, which can help create a visual distinction that can be trained in the algorithm and thus identify subjects regardless of their relative position (e.g., Ferreira et al. 2020). Particular care should be taken when using color-based methods that might rely on a specific mapping between a real-world color and the color values of the recorded pixels, as lighting, reflectance, and camera properties all play a role in how color is represented in the recorded video.

In our experience, manual annotation currently represents the best solution in those cases where automatic simultaneous tracking of multiple individuals is limited by specific characteristics of the model system. For very large datasets, semi-automatic tracking might be possible, with a human user able to re-label falsely assigned identifiers in post-processing (Pérez-Escudero et al. 2014).

### Available software and tools offered for video processing and analysis

As with recording equipment, the software available for video analysis ranges from free, open-source options to more expensive off-the-shelf or bespoke options (see Supplementary Table S2 for a non-exhaustive list of current popular options). When deciding which kind of software to use, time and financial budget need to be considered. Open-source solutions are by definition free of costs for software, but have associated hardware and maintenance costs. For automatic tracking, some approaches are extremely computationally intense and require either the purchase of dedicated processing hardware (GPUs) or of processing time on cloud computing platforms. Although the open-source community includes many tutorials and forums to help users (e.g., OpenBehavior.com or use search terms such as “open behavior”), researchers without extensive programming experience or IT support might consider using cloud computing services with pre-installed machine learning software. Sometimes this can save the considerable time and energy needed to install and maintain open source packages. In contrast, purchased software typically provides technical support and a more user-friendly interface, but at a higher financial cost.

### Post-processing of data/calibration

Regardless of the method used to track movements, the resulting output will be a set of pixel positions for each tracked video frame. The 2D image plane captured by the camera constitutes a single view of the 3D space in which the bird was moving. An optional next step, depending on the research question, is to transform the coordinate data into more meaningful units than pixels, or even into 3D coordinates. The final analysis of the coordinate data strongly depends on what is being investigated and we provide some simple examples below.

#### From image space to world coordinates

Some movements recorded in 2D, e.g., displacement of an individual, might need an additional step to translate the values into a more useful unit scale. Indeed, in field conditions, it is generally impossible for the focal individual to remain at the same distance from the camera, making it challenging to quantify their movement—a displacement of one pixel does not mean the same if the focal individual is 10 cm or 10 m away from the camera. To overcome this issue, movement can be quantified relative to the focal animal’s body length or height (Dudley 1990; Alexander et al. 1977; Walker 2004; Hein et al. 2012; Mitoyen et al. manuscript in preparation). For example, “the animal jumped 200% of its body length,” and not “the animal jumped 200 pixels.” Another possibility is to use a scale present in the image to translate a movement into objective units (Perez and Backwell 2020). Calibrating a movement is sometimes easier when filmed from above as movement in the vertical axis can be disregarded in many species. Provided that the camera stays in the same position and that a scaling object is available (either a scale or an object of known size), it is easy to calibrate movement and estimate precise distances and speed in laboratory conditions (Noldus et al. 2002; Crispim Junior et al. 2012; Chabert et al. 2016), but also in the field, for example, using GPS located drones (Raoult et al. 2018). To reconstruct 3D space, 2D video recordings from different perspectives can be combined using different types of calibration recordings (see “Camera calibration” in the “Case study: 3D motion capture in golden-collared manakins” section). Intrinsic calibrations can also be used for single-camera recordings to compensate for lens distortions, which are most obvious in recordings using wide-angle or “fisheye” lenses.

#### Analyzing coordinate data

The simplest quantity to calculate for two coordinates in the same frame, or one coordinate in two subsequent frames, is their displacement, for example, using Euclidean distance. The same approaches can be applied to 2D or 3D coordinates. For example, Mitoyen et al. (2021) calculated the vertical amplitude of the ring dove’s bowing behavior by subtracting the lowest bowing point of the tracked eye keypoint from the highest and obtained a result in pixels that was compared between males. Perez and Backwell (2020) calculated the amplitude of the waving display of fiddler crabs (*Austruca mjoebergi*) using the same approach, transforming it into millimetres using a scale present in the image. In courtship studies, coordinates defining the receivers’ movements may also be of interest, particularly cues signaling receptivity. Female bowerbirds solicit copulations by slowly crouching on the display arena, and a simple estimate of this movement can be obtained by measuring the vertical distance between head and feet key points and analyzing its change over time. Slow or rapid changes may be used as a correlate of receptivity or distress during courtship, respectively, both of which may predict changes in behavior in the courting male (Balsby and Dabelsteen 2002; Patricelli et al. 2002; Sullivan-Beckers and Hebets 2014; Spezie et al. manuscript in preparation). The line connecting two different keypoints in single frames can also be treated as a vector in polar coordinates to calculate angles or angular velocity; for example, allowing calculation of viewing angles relative to an object of interest in videos of an animal recorded from above.

More complex analysis is possible and depends on the particular research question, the number of tracked points per frame, and whether tracking estimates are available for the majority of recorded frames. For example, the 3D tracked jumps of male manakins described in the next section were used to estimate take-off velocity and force exerted during each jump (Janisch et al. 2021). To do so, Euclidean distance between frames was calculated and used to estimate speed, and the 3D movement sequences were segmented into jumps using a speed threshold. Each jump was projected from 3D to 2D space, and parabolic fits were used to estimate motion parameters including take-off angle and take-off velocity. This analysis approach can be used to investigate whether jump effort is associated with mating success.

## Case study: 3D motion capture in golden-collared manakins

In the last part of the manuscript, we illustrate an example application of a 3D motion capture system to study the elaborate courtship display of golden-collared manakins, a tropical Passerine bird that inhabits the rainforests of Panama and Colombia (Chapman 1935). Our group and collaborators have been studying the courtship behavior of this model species intensely over the last two decades (Fusani et al. 2007, 2014; [Bibr icab095-B18][Bibr icab095-B32]; Barske et al. 2015).

Manakins perform some of the most elaborate courtship displays in the animal kingdom. During the mating season, males gather in leks, where each of them possesses a courtship arena, and perform their display to attract females and gain matings (Chapman 1935). The courtship “jump-snap display” consists of a series of jumps between the saplings of the arena and ends with a “snap-grunt,” a cartwheel to the ground and a jump back to the sapling. Each jump is accompanied by a wingsnap, a loud sonation that males produce by colliding their modified wing bones over their back. The whole display is extremely rapid, to the point that it was only recently possible to study males’ movements in detail using high-speed video recordings (Fusani et al. 2007; Bodony et al. 2016). In a previous study, we found that the males' display is a rehearsed sequence adapted to their courtship arena (Janisch et al. 2020) and therefore spatial information plays a crucial role in their performance and for mating success. By using a 3D motion capture system, we wanted to investigate differences between males in greater detail while taking the spatial arrangement of their courtship arenas into account.

### Camera system and set-up

As we wanted to analyze 3D coordinates of manakin motion from field recordings, we used a custom-designed synchronized multi-camera recording system (Recnode, Loopbio GmbH, Austria), and a commercial software for automated tracking and 3D reconstruction (loopy, Loopbio GmbH, Austria). Cameras (Basler ace cameras, type: acA1920-155uc) were connected to a computer and a hardware synchronizer (Loopbio Trigger Box, Figure 2). We chose lenses (12.5 mm, LM12HC, Kowa Optical Products) for optimal depth of focus in the average arena diameter of 0.8–1.0 m. The system fit into a normal-sized backpack (47×34×15cm) for transportation.

In the field, we powered the system with a small portable 24 Volt car battery that lasted up to 3 h. With a wireless connection to the computer, we could change parameters such as exposure time and gain, and start and stop recordings from a distance of up to 8 m. Exposure time and gain are important features in a rapidly changing light environment and could be adjusted without affecting within-session calibration (see “Environmental challenges in field recordings” section).

### Recordings

We recorded courtship displays from three cameras at 60 fps and a resolution of 1,920×1,200 pixels. This was the optimum recording quality for capturing the movement of interest with good spatial and temporal resolution and reasonable file size, considering that we were interested in the motion of the jumping bird and not in viewing any specific body parts (see “Size of species and level of spatial detail needed” and “Speed of movements” sections). Cameras were synchronized using a hardware synchronizer (see section “Simultaneous multi-device recordings”). Before each recording session, we positioned the cameras on gorilla pods or tripods at different heights around the bird’s arena at a distance of 1–3 m, and set the aperture and focus manually on the lens of each camera. We removed any small branches and leaves that occluded the cameras’ views of the court.

### Camera calibration

Camera calibrations refer to the acquisition of precise knowledge about the optics (aperture, distortion of the lenses) of the cameras and their positions relative to each other (Jackson et al. 2016). Therefore, every time camera positions and lens settings were set or changed, we recorded calibration videos. It is advisable to do a quality check of calibration videos as soon as possible after recording. Failing calibrations will prevent all video material associated with those calibration videos from being used for later 3D analysis (see “Pre-screening of video recordings” section). We performed intrinsic, extrinsic, and ground-level calibrations. Intrinsic calibration corrects the distortion of the lens for each camera separately, extrinsic calibration collocates the cameras within the same 3D space, and ground-level calibration ensures that the common 3D space is oriented correctly relative to the real world. All three are equally important for acquiring 3D recordings from multi-camera videos. For extrinsic calibrations, a frame rate of 30 fps, and for intrinsic calibrations and ground-level calibrations 10 fps were sufficient. Extrinsic and ground-level calibration videos must be recorded with synchronized cameras.

So far, several methods and algorithms have been developed for camera calibrations (reviewed by Hartley and Zisserman 2004). In our case, we used a chequerboard for intrinsic calibration, which had to be presented to each camera at many possible angles and distances. For extrinsic calibration, we used two wands with colored LEDs at the ends, one with blue lights and the other with red lights. We repeated extrinsic calibration videos with both lights to be sure to find the best color to be tracked for the particular light conditions. For ground-level calibration, we had a multi-armed structure with fixed points at known distances, and a spirit level to allow precise positioning relative to horizontal (Model QF-26, Firefly Instrument (HK) Co Ltd., Beijing, Figure 2D). We placed it leveled in the center of the arena so that at least five fixed points could be visible in each camera. Sometimes, we were unable to perform a ground-level calibration with the multi-armed structure due to weather conditions or small arenas. For ground-level calibration, at least five fixed points with known real-world coordinates are needed, with a maximum of four being co-planar (Jackson et al. 2016). We could, therefore, use five fixed points on the saplings of the arenas, and used the distances between the saplings to calculate the 3D positions of the five points. A comparison of the two used ground-level calibrations showed similar and reliable results. Finally, in one case, the courtship arena of a male was on an incline and therefore we added 0.1 m to all *z*-values of the points of the multi-arm structure to “lift” space and prevent negative *z*-values. Otherwise, it seemed that the bird was jumping underground which would have caused difficulties for further analysis.

### Automated object tracking in 3D

Camera calibration videos were post-processed with the software Loopy (Loopbio GmbH, Austria) for automated tracking and subsequent 3D reconstruction of a bird’s movement. In this study, we used the entire collection of manual annotations of male courtship videos from a previous field season (150,000 frames) to train the machine learning algorithm to detect the bird without using markers. Annotations were made of the entire bird using a bounding box (see “Different aspects of motion analysis: object detection, keypoint estimation, pose detection” section). After acquiring automated tracking results for each 2D recording, we used calibrations to combine tracked videos to reconstruct the 3D space (see “From image space to world coordinates” section). The final output of automated tracking was text files containing *x*, *y*, and *z* coordinates of the tracked bird’s movement for each frame of the video. Subsequent analysis of these coordinates was used to estimate parameters of motion for each jump of each male, including take-off velocity and angle, jump curvature, and total jump speed (Janisch et al. 2021).

## Conclusions

Here we have discussed different applications of video recordings and motion tracking in the context of quantifying movements to better understand avian courtship. New technologies allow us to address and answer research questions from novel perspectives. For example, particular movements in a courtship display can provide information about different aspects of male quality (vigor versus skill) and allow us to study whether they undergo sexual selection and play a role in female choice (Janisch et al. 2020, 2021).

An alternative to using video to capture movements is the use of bio-loggers such as gradiometers/accelerometers. These devices can provide a higher sampling rate than video, but only record data from the single viewpoint of the tracking device. As it might be impossible to place such devices on the precise body part of interest, or indeed on multiple body parts, we believe that video recordings are essential for describing the movements involved in courtship. This also applies to many other animal behaviors. We have shown that video recordings require considerable planning, as many different aspects have to be considered. Although time-consuming, collecting high-quality video material should be considered a good investment. Video analysis techniques are rapidly developing and may allow us to answer open research questions by applying new technologies to previously recorded videos.

We emphasized in the section “Post-Processing video material you have collected” that manual annotations of movements may be sufficient, depending on the precise research question. Training of automated tracking algorithms can be time-intensive, and includes the careful selection of representative training data. Overall, machine learning has great potential for the analysis of large sets of complex data needed to answer contemporary questions in animal behavior (Mathis et al. 2020). This is also true for the rising field of unsupervised learning approaches (Khanum et al. 2015). In addition, there is an interesting further application of the automated tracking of animal movements, namely to use machine learning to classify movement patterns into behavior. Ethologists have traditionally invested huge amounts of time into manual scoring or coding of behavior in video recordings. In theory, coordinate movement data from video sequences that have been manually scored for different behaviors could be used to train algorithms to recognize particular behaviors in motion sequences. The new field of computational ethology (Anderson and Perona 2014) may make it easier to quantify behavior, and we hope that it is clear that there are many aspects to be considered in terms of data collection and video analysis.

## Statement on human and animal rights

All procedures in the manakin case study were authorized by the Smithsonian Tropical Research Institute and the Autoridad Nacional del Ambiente of the Republic of Panama (No. SE/A-118-17, 28th December 2017).

## Funding

This work was supported by several funds including start-up funds to L.F. by the University of Vienna and the University of Veterinary Medicine, Vienna as well as grants from the Austrian Science Fund (FWF) [W1262-B29], and Vienna Science and Technology Fund (WWTF) [CS18-021]. J.J. received a travel grant (KUWI MA) from the University of Veterinary Medicine, Vienna. This material is based upon work supported by the National Science Foundation under grant number 1457541.

## Supplementary Material

icab095_Supplementary_Data

## Notes

From the symposium “Manakin genomics: comparative studies of evolution and behavior in a unique clade of birds” presented at the virtual annual meeting of the Society for Integrative and Comparative Biology, January 3–7, 2021.

The authors C. M., E. P., and G. S. contributed equally to this work.
