# Supplementary material for: Video Recording and Analysis of Avian Movements and Behavior: Insights from Courtship Case Studies
Source: Integr Comp Biol. 2021 May 25;61(4):1378–93. doi: 10.1093/icb/icab095 (PMC8516111; doi:10.1093/icb/icab095)
Supplement: icab095_Supplementary_Data [file icab095_supplementary_data.zip › icb-2021-0070-File005.pdf]

Supplemental Table 1. Different kinds of cameras that can be used to record video for later analysis of animal movements.

Type	Summary	Pros	Cons
**Stand-alone cameras**			
Action cam	Small, lightweight, robust, portable cameras for outdoor sports enthusiasts. High-definition quality & high frame rates. Recordings stored on exchangeable miniSD cards. Exchangeable batteries.	Relatively cheap and easily available; wide range of mounting accessories; robust to environmental challenges of field work.	Fixed focus lens.
Camcorder	Portable camera designed for video recording. Recordings stored on internal memory or SD cards. Battery or mains power usually possible. Newer models more robust.	Some models have optical zoom; usually mountable on tripods; usually have autofocus and autoexposure settings.	Bulkier than action cameras.
Camera trap	Typically fixed in position for longer periods. Recording triggered by motion sensor.	Least disturbance to animals because of minimal human interaction.	Hardware problems undetected if camera not regularly checked. False triggers result in unwanted recordings.
Digital SLR camera	Modern single lens reflex cameras. High-definition video. Professional level cameras with high user control over lens and camera settings.	More user control over settings; interchangeable lenses; better results than camcorder in low light.	Fewer automatic setting options; experienced camera operators get best results.
**Streaming cameras** (computer required)			
Webcam	Small, streams video through computer network.	Relatively cheap; suitable for long recordings.	File sizes constrained by streaming, video may be low resolution / low frame rate / compressed
USB camera	Small, transmits video via USB to recording computer, used in industrial applications.	Interchangeable lenses; wide range of sensors, resolutions, frame rates available.	Computer required to control recording and store data. Can be expensive depending on hardware specifications.
GigE vision	Industrial camera standard for high-speed streaming via ethernet.	Interchangeable lenses; streaming quality maintained over longer cables than USB (to 100m).	Computer required to control recording and store data. Can be expensive depending on hardware specifications.

Supplemental Table 2. Non-exhaustive list of currently available open-source software that can be used to analyse video material containing animal movements. The name, website, method (CV: computer vision; ML: machine learning), language are listed, and whether or not the software performs individual identification (ID), motion detection (MD), object detection (OD), keypoint tracking (KP), pose estimation (PE), deals with multiple individuals (MI), and whether it performs behavioural classification (BC). None of the listed software requires the animals to be marked in a particular way. References are listed below.

Name	Link	Method	Language	ID	MD	OD	KP	PE	MI	BC
DeepPoseKit	https://github.com/jgraving/deepposekit	ML	Python	no	no	no	yes	yes	no	no
DeepLabCut	https://github.com/DeepLabCut/DeepLabCut	ML	Python	no	no	no	yes	yes	yes	no
SLEAP	https://sleap.ai/	ML	Python	no	no	no	yes	yes	yes	no
JAABA	http://jaaba.sourceforge.net/	ML	Matlab	no	no	no	no	no	yes	yes
B-SoiD	https://github.com/YttriLab/B-SOID	ML	Python	no	no	no	no	no	no	yes
idTracker	http://www.idtracker.es/	CV	Matlab	yes	no	yes	no	no	yes	no
idtracker.ai	http://idtracker.ai/	CV+ML	Python	yes	no	yes	no	no	yes	no
MotionMeerkat	https://github.com/bw4sz/OpenCV_HummingbirdsMotion	CV	Python	no	yes	no	no	no	no	no
TRex	https://trex.run/	CV+ML	Python	yes	no	yes	no	yes	yes	no
Simba	https://github.com/sgoldenlab/simba	ML	Python (Windows)	no	no	no	no	no	yes	yes

References:

DeepPoseKit: Graving, J. M., Chae, D., Naik, H., Li, L., Koger, B., Costelloe, B. R., & Couzin, I. D. (2019). DeepPoseKit, a software toolkit for fast and robust animal pose estimation using deep learning. *Elife*, 8, e47994. doi: 10.7554/eLife.47994

DeepLabCut: Mathis, A., Mamidanna, P., Cury, K. M., Abe, T., Murthy, V. N., Mathis, M. W., & Bethge, M. (2018). DeepLabCut: markerless pose estimation of user-defined body parts with deep learning. *Nature neuroscience*, 21(9), 1281-1289. doi: 10.1038/s41593-018-0209-y

SLEAP: Pereira, T. D., Tabris, N., Li, J., Ravindranath, S., Papadoyannis, E. S., Wang, Z. Y., Turner, D.M., McKenzie-Smith, G., Kocher, S.D., Falkner, A.L., Shaevitz, J.W. & Murthy, M. (2020). SLEAP: multi-animal pose tracking. *bioRxiv*. doi: 10.1101/2020.08.31.276246

JAABA: Kabra, M., Robie, A. A., Rivera-Alba, M., Branson, S., & Branson, K. (2013). JAABA: interactive machine learning for automatic annotation of animal behavior. *Nature Methods*, 10(1), 64. doi: 10.1038/nmeth.2281

B-SoiD: Hsu, A. I., & Yttri, E. A. (2021). An Open Source Unsupervised Algorithm for Identification and Fast Prediction of Behaviors. bioRxiv, 770271. doi: 10.1101/770271

idTracker: A. Pérez-Escudero, J. Vicente-Page, R.C. Hinz, S. Arganda, G.G. de Polavieja (2014). *Nature Methods* 11(7):743-748. doi: 10.1038/nmeth.2994

idtracker.ai: Romero-Ferrero, F., Bergomi, M. G., Hinz, R. C., Heras, F. J., & de Polavieja, G. G. (2019). Idtracker.ai: Tracking all individuals in small or large collectives of unmarked animals. *Nature Methods*, 16(2), 179-182. doi: 10.1038/s41592-018-0295-5

MotionMeerkat: Weinstein, B. G. (2015). MotionMeerkat: integrating motion video detection and ecological monitoring. *Methods in Ecology and Evolution*, 6(3), 357-362. doi: 10.1111/2041-210X.12320

TRex: Walter, T., & Couzin, I. D. (2021). TRex, a fast multi-animal tracking system with markerless identification, and 2D estimation of posture and visual fields. *Elife*, 10, e64000. doi: 10.7554/eLife.64000

Simba: Nilsson, S. R., Goodwin, N. L., Choong, J. J., Hwang, S., Wright, H. R., Norville, Z., Tong, X., Lin, D., Bentzley, B.S., Eshel, N., McLaughlin, R.J., & Golden, S. A. (2020). Simple Behavioral Analysis (SimBA): an open source toolkit for computer classification of complex social behaviors in experimental animals. *BioRxiv*. doi: 10.1101/2020.04.19.049452

Supplemental Videos:

SupplVideo1_Bowerbird.mp4: Sample video recorded at Taunton National Park, Queensland, Australia, 2018. Coloured annotations are the result of automatic tracking of multiple keypoints on two spotted bowerbirds during a courtship interaction. Different labels are consistently assigned to the courting bird and to the recipient, indicated by 1 or 2 after the label name, respectively. Note also that left and right labels are correctly assigned.

SupplVideo2_Dove.mp4: Sample video recorded in the Dove Lab, University of Vienna, 2018. Coloured annotations are the result of keypoint detection on a male ring dove during a courtship display. The detection successfully tracks the tip of the tail (tail_tip), feet (foot_left, foot_right), eyes (eye_right; eye_left is occluded) and beak (keypoint: beak_tip) of the bird.

SupplVideo3_Manakin.mp4: Sample video recorded in Panama, 2018. Red points show automated object tracking of a male golden-collared manakin during his courtship display, detecting the centre of the body.

SupplVideo4_Peguin.mp4: Sample video recorded on Kerguelen Island, 2020. Coloured labels show automated keypoint detection of a courting penguin pair tracked together using keypoint detection. Different labels (for example: beaktip and beaktip2) were used to distinguish between the female and the male.

See Table 1 in the main text for more details on the related case studies.
